# What topics are women interested in during pregnancy: exploring the role of social media as informational and emotional support

**DOI:** 10.1186/s12884-022-04842-5

**Published:** 2022-06-26

**Authors:** Joo Yun Lee, Eunhee Lee

**Affiliations:** 1 College of Nursing, Gachon University, Incheon, Korea; 2 School of Nursing, Hallym University, 1 Hallymdaehak-gil, Chuncheon, Gangwon-do Korea

**Keywords:** Social networking, Social media, Consumer health information, Information seeking behavior, Pregnant women

## Abstract

**Background:**

Social media plays an important role as a source for obtaining or sharing health information. It is frequently used as a resource by pregnant women who seek information and emotional support regarding their physical and role changes. To provide high-quality support for pregnant women, it is important to determine what topics they want information on from social media during their pregnancy, and what effects they expect from each topic. This study investigates what topics pregnant women are interested in according to their stage of pregnancy and need for informational or emotional support from each topic of interest.

**Methods:**

An online survey was conducted with 302 pregnant Korean women. The survey questionnaires included information sources and experience of social media. Along with the questionnaires, they were asked to describe three to ten health topics that they were most interested in in the last two weeks.

**Results:**

Social media (72.85%) and search engines (72.85%) were found to be most frequently used for pregnancy-related health information. The topics of interest slightly differed by stage, but mainly postpartum care, pregnancy symptoms, and daily life issues ranked high. Mood related topics appeared frequently among women in their first trimester or post-partum stage of pregnancy. Informational support was mostly needed for daily life issues, and emotional support needs centered mostly around stories about family and mood during pregnancy.

**Conclusions:**

Pregnant women have different needs for informational or emotional support, depending on their stage of pregnancy and their topics of interest. Therefore, social media support should be delivered with varying content and platforms tailored to women’s needs at each trimester.

**Supplementary Information:**

The online version contains supplementary material available at 10.1186/s12884-022-04842-5.

## Background

It is well known that both the public and patients actively use the Internet to obtain health-related information. Constant availability, anonymity, ease of access, and support that overcomes space constraints have been identified as advantages of obtaining information through the Internet [1].

Among the various channels (e.g., search engines, instant messaging, and apps) that can be used to obtain health information from the Internet, social media has recently attracted attention. Social media has the advantage of gathering a large number of people with the same interest or concern [2] to directly create information, rather than just obtaining it [3–5]. Therefore, information can be actively exchanged, and in this process, participants voluntarily exchange information and emotional support [5, 6].

According to Pew Internet reports [7, 8], among the health information topics delivered online, food, drug safety, and pregnancy-related health information were ranked highest. Although pregnancy is a normal life cycle process and not a disease, many women feel anxiety about unfamiliar physical and role changes. Therefore, they need information and emotional support to help them make decisions related to pregnancy and to relieve anxiety [9, 10].

Health-related information needs include the demand for expertise-based information from health professionals and experience-based information from people in similar conditions [2, 11]. Social media is a very useful source of information for pregnant women because it has accumulated a large number of user-generated experiences. In the process of communicating through social media, pregnant women gain a sense of relief that they are connected to the world [12] and can reduce stress by expressing their emotions [5]. This is the emotional support that pregnant women receive from social media.

Despite such useful information and emotional support, most pregnant women rate the credibility of information obtained through online social media lower than that obtained from health professionals and judge the reliability of every piece of information based on their own subjective criteria [3, 4]. Therefore, in the new wave of technology, it is necessary to deliver more reliable information to pregnant women [4]. An example would be the combination of high-quality information from experts and warm social media support [13]. It is expected that the effectiveness of social media will increase when topical information and emotional support suitable for the stage of pregnancy are provided.

To provide appropriate support for pregnant women, it is important to determine the topics that they want from social media and the effects they expect from each topic. Studies on the pregnant women’s information needs was mainly conducted as qualitative research [3, 9] or focused on what kind of online sources were used [12, 14]. No studies have been conducted on topics required by stage of pregnancy. Thus, this study aims to identify the needs of pregnant women for providing effective support, focusing on what topics pregnant women are interested in by stage of pregnancy.

## Methods

### Aim of study

The aim of this study is as follows: 1) to investigate what information sources pregnant women frequently use to obtain pregnancy-related health information, especially how much social media they use; 2) to identify what topics pregnant women are interested in by stage of pregnancy; and 3) to compare satisfaction with informational support or emotional support by topic.

### Participants

An online survey was conducted in May 2021, with a link to the online research notice being sent to the panelists registered with Gallup, a leading survey research company in Korea. Subsequently, more participants were recruited using snowball sampling. Participants were limited to women who were pregnant or within one month of childbirth. At the beginning of the survey, questions such as the number of weeks of pregnancy, expected date of confinement, and hospital information was asked to confirm participant’s eligibility. About 267 or more participants were needed to have a confidence level of 95% with a margin of error is within ± 6% of the surveyed value. In addition, in order to minimize selection bias due to snowball sampling, we continued to recruit with the aim of collecting at least 30 samples from each trimester group. As a result, a total of 302 participants were recruited.

### Questionnaire and measurement

The researchers designed the survey questionnaires based on previous studies [14, 15] including demographics, information sources, interested topics, and experience of social media. The options for preferred information sources provided in the questionnaire were health professionals, family, friends, medical-related websites (such as hospital or health center websites, or government portals), childcare-related commercial websites (such as baby milk formula companies), social media (such as online communities, blogs, YouTube, Facebook, Instagram, or Twitter), search engines (such as Google, Naver, or Daum), books, newspapers, and TV programs. The participants were able to select all relevant items. Additionally, they were asked to describe three to ten health topics that they were most interested in during the preceding two weeks.

Subsequently, the participants were divided into those who used social media as a health information source and those who did not. Social media users were asked how much information and emotional support was received from each topic and indicate on a 5-point scale ranging from “1 point = not satisfied” to “5 point = very satisfied” what their level of satisfaction was with the support they received. Then, they were asked how often they used social media and the reasons for using social media as a source of health information.

All participants’ digital literacy was measured. The eHealth Literacy Scale (eHEALS) measures consumers’ knowledge, comfort, and perceived skills when using online information for health [16]. In this study, KeHEALS, which was developed by Chang et al. as a Korean version through the cultural adaptation process of eHEALS, was used [17]. This tool consists of ten questions. The first two questions are used to understand the respondents’ interest in using online health information and are not included in the scoring process. The eight items included in the score calculation process, reflecting the ability to understand online health information, were measured using a 5-point Likert scale. The total scores for the eight items range from 8–40 points, with a higher score indicating a higher ability to understand online health information. In the development study, the reliability measured with Cronbach's ⍺ was 0.88 and 0.70 for eHEALS and KeHEALS, respectively. In terms of validity, the content validity of KeHEALS was found to be 1.0. The construct validity was verified by exploratory factor analysis, confirmatory factor analysis, and hypothesized relationship testing. The total score of the KeHEALS was significantly correlated with the attitudes toward internet health information. Cronbach's ⍺ in this study was 0.87. This study was conducted after obtaining permission from the researchers of eHEALS and KeHEALS.

### Analysis

All items were analyzed using descriptive statistics, including frequencies and percentages. A *t*-test and chi-square test (Fisher’s exact test) were performed to compare the characteristics of the social media user group and non-user group. The difference of social media usage time by pregnancy stage was analyzed by ANOVA, followed by Scheffe test for multiple group comparison. The responses collected in the narrative form for “the topics of interest in the past two weeks” were categorized based on Lee [18] study, and the frequency of each category was measured.

### Ethical considerations

The institutional review board of Gachon University, Incheon, Korea (No. 2020–207) approved this study. A research notice, consent form, and online questionnaire were developed with the help of Gallup and an access link or QR code was sent to the panelists. The research notice explained the purpose of the study, the contents of the questionnaire, and the guarantee of confidentially to the research participants as well as their right to withdraw from the study at any time if they so wish. When participants expressed their intention to participate in the study, they completed an online informed consent form, and a survey was conducted. The final data were anonymized by Gallup.

## Results

### Demographics

The average age of the 302 pregnant women who participated in the study was 32.86 ± 3.56 years with 30–34 years (153, 50.7%) being the most common. There were 208 primigravidas (68.9%) and 40 women (13.2%) in their first trimester, 113 in the second (37.4%), 73 in the third (24.2%), and 76 in the postpartum period (25.2%). Their mean KeHEALS score was 31.00 ± 4.09 (Table 1). Among them, 220 women (72.8%) used social media, and 82 (27.2%) did not. The KeHEALS score of the social media user group was 31.29 ± 4.24, which was higher than that of the non-user group (*p* = 0.041). No statistical differences were found between the two groups in terms of other pregnancy characteristics.

**Table 1:** Characteristics of participants

		Total				Use (*n* = 220)		Do not use (*n* = 82)		chi or t(p)	
		n(%)		Mean ± SD		n(%) or Mean ± SD		n(%) or Mean ± SD			
Use of social media	Use	82	(27.2)								
	Do not use	220	(72.8)								
Age (years)	20–24	3	(1.0)	32.86	± 3.56	32.79	± 3.44	33.04	± 3.87	.533	(.597)
	25–29	46	(15.2)								
	30–34	153	(50.7)								
	35–39	88	(29.1)								
	40–44	12	(4.0)								
Number of pregnancies	1^st^ (primipara)	208	(68.9)			146	(66.4)	62	(75.6)	2.251^a^	(.335)
	2^nd^	80	(26.5)			63	(28.6)	17	(20.7)		
	3^rd^ and above	14	(4.6)			11	(5.0)	3	(3.7)		
Fertility treatment	No	266	(88.1)			73	(89.0)	193	(87.7)	1.182^a^	(.574)
	Intrauterine Insemination	11	(3.6)			4	(4.9)	7	(3.2)		
	In Vitro Fertilization	25	(8.3)			5	(6.1)	20	(9.1)		
Stage	1^st^	40	(13.2)			31	(14.1)	9	(11.0)	4.746	(.200)
	2^nd^	113	(37.4)			82	(37.3)	31	(37.8)		
	3^rd^	73	(24.2)			58	(26.4)	15	(18.3)		
	postpartum	76	(25.2)			49	(22.3)	27	(32.9)		
KeHEALS	< 30	95	(31.5)	31.00	± 4.09	31.291	± 4.238	30.207	± 3.589	-2.056	(.041)
	≥ 30	207	(68.5)								

^a^ Fisher’s exact test; KeHEALS, Korean eHealth literacy scale

### Information source and interested topics from all participants

Social media (220, 72.8%) and search engines (220, 72.8%) were found to be the most frequently used sources of pregnancy-related health information when items were available for multiple selection. This was followed by friends (180, 59.6%), health professionals (149, 49.3%), and family (115, 38.1%). The social media user and non-user groups had the same priority of information source use, except for social media (Table 2).

**Table 2:** Sources of health information related to pregnancy (multiple selection)

Sources of health information	Total (*n* = 302)		Use social media (*n* = 220)		Do not use social media (*n* = 82)	
	n	(%)	n	(%)	n	(%)
Social media (including online communities, YouTube, Facebook, Instagram, and Twitter)	220	(72.9)	220	(100.0)	0	(0.00)
Search engine (including Naver, Daum, and Google)	220	(72.9)	176	(80.0)	44	(53.7)
Friends	180	(59.6)	138	(62.7)	42	(51.2)
Health professionals	149	(49.3)	119	(54.1)	30	(36.6)
Family	115	(38.1)	89	(40.5)	26	(31.7)
Books and newspapers	80	(26.5)	67	(30.5)	13	(15.9)
Medical-related websites (such as hospitals, health centers or government portals)	66	(21.9)	49	(22.3)	17	(20.7)
TV program	49	(16.2)	41	(18.6)	8	(9.8)
Childcare-related commercial websites (such as baby milk formula companies)	37	(12.3)	31	(14.1)	6	(7.3)
Others	3	(1.0)	3	(1.4)	0	(0.0)

Table 3 shows the topics of interest according to pregnancy stage. In the first trimester, pregnancy symptoms (such as morning sickness), daily life during pregnancy (such as travel or exercise), and hospital information frequently appeared. In the second trimester, it was pregnancy symptoms (such as uterine cramping), fetus-related information (such as sex or fetal movement), and postpartum care, in the third trimester, it was pregnancy symptoms (symptoms indicating childbirth), postpartum care, and daily life during pregnancy (such as exercise for childbirth or diet) that frequently appeared. In the postpartum period, it was postpartum care and daily life during the postpartum period (such as diet with breast feeding or diet for recovery). The social media non-user group had a higher interest in hospital information than the user group (Table 4).

**Table 3:** Interested topics by stages

Topics	**1st**		**2nd**		**3rd**		**Postpartum**		**Total**	
	n	(%)	n	(%)	n	(%)	n	(%)	n	(%)
Post-partum care	20	(50.0)	56	(49.6)	46	(63.0)	48	(63.2)	170	(56.3)
Pregnancy symptoms (such as morning sickness, itching or uterine cramping)	31	(77.5)	60	(53.1)	51	(69.9)	22	(28.9)	164	(54.3)
Daily life issues (such as diet, exercise, work or travel)	25	(62.5)	55	(48.7)	36	(49.3)	31	(40.8)	147	(48.7)
Fetal development (such as sex or fetal movements)	16	(40.0)	58	(51.3)	26	(35.6)	16	(21.1)	116	(38.4)
Antenatal investigations and screening (such as testing for gestational diabetes or USG)	18	(45.0)	53	(46.9)	20	(27.4)	9	(11.8)	100	(33.1)
Hospital information (such as choosing a hospital)	22	(55.0)	37	(32.7)	12	(16.4)	28	(36.8)	99	(32.8)
Medications (such as iron or vitamins)	19	(47.5)	36	(31.9)	12	(16.4)	16	(21.1)	83	(27.5)
COVID-19	4	(10.0)	26	(23.0)	13	(17.8)	25	(32.9)	68	(22.5)
Mood during pregnancy	12	(30.0)	16	(14.2)	8	(11.0)	17	(22.4)	53	(17.6)
Stories about family (such as changes in family roles)	1	(2.5)	11	(9.73)	9	(12.3)	23	(30.3)	44	(14.6)
Others	0	(0.0)	0	(0.0)	1	(1.37)	0	(0.0)	1	(0.3)
Totals of each stage	40	(100.0)	113	(100.0)	73	(100.0)	76	(100.0)	302	(100.0)

**Table 4:** Comparison of user and non-user groups regarding interested topics

Topics	**Use social media (*n* = 220)**								**Do not use social media (*n* = 82)**							
	1st (*n* = 31)		2nd (*n* = 82)		3rd (*n* = 58)		Postpartum (*n* = 49)		1st (*n* = 9)		2nd (*n* = 31)		3rd (*n* = 15)		Postpartum (*n* = 27)	
	n	(%)	n	(%)	n	(%)	n	(%)	n	(%)	n	(%)	n	(%)	n	(%)
Post-partum care	16	(51.6)	45	(54.9)	38	(65.5)	33	(67.4)	4	(44.4)	11	(35.5)	8	(53.3)	15	(55.6)
Pregnancy symptoms (such as morning sickness, itching, or uterine cramping)	26	(83.9)	51	(62.2)	42	(72.4)	19	(38.8)	5	(55.6)	9	(29.0)	9	(60.0)	3	(11.1)
Daily life issues (such as diet, exercise, work, or travel)	20	(64.5)	45	(54.9)	27	(46.6)	27	(55.1)	5	(55.6)	10	(32.3)	9	(60.0)	4	(14.8)
Fetal development (such as sex or fetal movements)	13	(41.9)	46	(56.1)	22	(37.9)	13	(26.5)	3	(33.3)	12	(38.7)	4	(26.7)	3	(11.1)
Antenatal investigations and screening (such as testing for gestational diabetes or USG)	16	(51.6)	50	(61.0)	19	(32.8)	7	(14.3)	2	(22.2)	3	(9.7)	1	(6.7)	2	(7.4)
Hospital information (such as choosing a hospital)	18	(58.1)	21	(25.6)	11	(19.0)	15	(30.6)	4	(44.4)	16	(51.6)	1	(6.7)	13	(48.1)
Medications (such as iron or vitamins)	16	(51.6)	22	(26.8)	11	(19.0)	5	(10.2)	3	(33.3)	14	(45.2)	1	(6.7)	11	(40.7)
COVID-19	3	(9.7)	17	(20.7)	10	(17.2)	8	(16.3)	1	(11.1)	9	(29.0)	3	(20.0)	17	(63.0)
Mood during pregnancy	10	(32.3)	13	(15.9)	7	(12.1)	13	(26.5)	2	(22.2)	3	(9.7	1	(6.7)	4	(14.8)
Stories about family (such as changes in family roles)	1	(3.2)	9	(11.0)	8	(13.8)	14	(28.6)	0	(0.0)	2	(6.5)	1	(6.7)	9	(33.3)
Others	0	(0.0)	0	(0.0)	1	(1.7)	0	(0.0)	0	(0.0)	0	(0.0)	0	(0.0)	0	(0.0)

### Experience of social media use from user group

The level of satisfaction obtained through social media by topic is shown in Table 5. Informational satisfaction was high in “daily life during pregnancy,” “antenatal exams,” and “family related” and “symptom-related” topics. Emotional satisfaction was high in “story about family,” “mood during pregnancy,” “daily life,” and “symptoms” (in that order).

**Table 5:** Level of satisfaction regarding information and emotional support by topics

Topics	Information support			Emotional support		
	**Mean**	** ± SD**	**Rank**	**Mean**	** ± SD**	**Rank**
Daily life issues	4.025	± 0.838	1	4.109	± 0.909	3
Antenatal investigations and screening	4.000	± 0.852	2	4.054	± 0.965	5
Stories about family	3.906	± 0.963	3	4.313	± 0.859	1
Pregnancy symptoms	3.870	± 0.995	4	4.065	± 0.939	4
Mood during pregnancy	3.767	± 1.109	5	4.140	± 1.037	2
Fetal development	3.766	± 1.031	6	4.053	± 0.896	6
Post-partum care	3.750	± 1.051	7	3.886	± 0.986	7
Hospital information	3.662	± 1.094	8	3.831	± 1.009	8
Medications	3.574	± 0.964	9	3.778	± 0.945	9
COVID-19	3.447	± 1.267	10	3.526	± 1.133	10

Members of the social media user group accessed social media to obtain pregnancy information an average of 21.94 ± 22.94 times a week, and commented on other people’s posts (10.75 ± 15.22 times in a week) more frequently than writing new posts (6.62 ± 10.79 times in a week). The total number of times of using social media was highest in the postpartum period, and the third trimester was higher than the first and second trimester (Table 6).

**Table 6:** Frequency of using social media

Use of social media	Total	1st^a^	2nd^b^	3rd^c^	Postpartum^d^	F(p) and scheffe
	Mean ± SD	Mean ± SD	Mean ± SD	Mean ± SD	Mean ± SD	
Times to access social media (in a week)	21.94 ± 22.94	14.94 ± 9.12	18.87 ± 19.75	21.17 ± 17.26	32.41 ± 34.30	5.154 (.002) d > a,b,c
Times to write a post (in a week)	6.62 ± 10.79	3.65 ± 5.36	3.61 ± 5.54	5.59 ± 9.85	14.78 ± 15.99	14.768 (< .001) d > a,b,c
Times to write a comment on the post (in a week)	10.75 ± 15.22	8.36 ± 11.80	7.31 ± 10.44	9.22 ± 16.09	19.82 ± 19.15	8.429 (< .001) d > a,b,c

Participants found social media to be more useful than other sources of information and indicated the following as their reasons for its use: they could quickly obtain information (88.2%), obtain new information (78.2%), and receive emotional support from other users (76.8%) (Table 7). In some free-text answers, it was mentioned that information on social media with many subscribers and a large number of accumulated posts is useful. It was noted that it is possible to obtain information about unusual and rare experiences or general situations that many people experience.

**Table 7:** Reasons for using social media as a source of health information (multiple selection)

Reasons	**n**	**(%)**
To get information quickly (rapidly)	194	(88.2)
To get new information	172	(78.2)
To get emotional support through relatable experiences	169	(76.8)
To get applicable information	147	(66.8)
To get detailed information	135	(61.4)
To check information before and after seeing your doctor	118	(53.6)
To have fun through the relatable stories of pregnant women	104	(47.3)
To get reliable information	58	(26.4)
Other	3	(1.4)

## Discussion

This study conducted a survey to identify why pregnant women use social media as a source for health information, the topics they want to obtain information about according to their stage of pregnancy, and the effects of obtaining support from social media.

### Social media as information source during pregnancy

In this study, 72.8% of participants used social media for pregnancy-related health information. Social media and search engines ranked highest in the same rankings, followed by friends (59.6%), and health professionals (49.3%). According to a study by Lupton and Maslen [11] that focused on Australian women’s use of digital technologies for health (not limited to specific health problems), healthcare providers and online sources were the most used health information sources. The most frequently used online sources were search engines (100%), whereas social media accounted for 33% of use. The results of our study showed that the use of social media was higher than that in the Lupton and Maslen [11], which might be attributed to the characteristic of Korean who would be more likely to trust and use social internet sites [2]. No previous studies have numerically compared the use of social media related to pregnancy, but many studies have confirmed women’s dependence on social media and the positive effects of social media use during pregnancy [3, 12, 19]. In a study comparing the United States, Korea, and Hong Kong for the use of health information sources [2], Korea showed the highest internet use for health information. Compared to the United States, which frequently uses online professional health sites, Korea uses blogs, and Hong Kong frequently used social networking sites. This can support the finding of high social media use by pregnant Korean women in the current study.

In this study, the demographic difference between the social media user and non-user groups appeared only in eHEALS, an indicator of the ability to use online information for health. Lupton and Maslen [11] stated that women’s digital health use was not related to education level or region but was more related to age, whether they had children, or whether they had family members with health problems. It can be interpreted that the social media use rate of women going through the experiences of pregnancy and childbirth is high, regardless of demographic characteristics.

In the question of why participants use social media, “to get information quickly,” “to get new information” or “to get applicable information,” and “to get emotional support” were ranked high. Chuang and Yang [5] analyzed the effects of social media as informational support and nurturant support and emphasized the importance of both for addressing health problems. The patterns of information and nurturant support differed depending on health topics or communication formats. In this study, both forms of support were confirmed as important for pregnant women.

The advantages of obtaining information through social media are: online information can be accessed quickly and easily compared to consultation with a health professional, and it provides a huge amount of detailed information compared to books and educational materials. Pregnant women feel that social media as an information source helps them in their personal decision-making process [20]. In addition, because pregnant women feel that asking healthcare professionals questions during appointments would bother them, or feel that communication with healthcare professionals is improved if they are informed in advance, they gather information in advance through social media [9, 15]. During pregnancy, positive emotions can be felt while sharing experiences with other women in similar situations through social media (reassurance), assuring yourself that you are not alone, and confirming that there is no problem with fetal development (normalized) [14]. Particularly, social media is more attractive to users because they can both create and obtain information. By writing new posts or exchanging comments, pregnant women feel connected to others and reduce anxiety about being isolated due to pregnancy [12, 21]. Additionally, it was found that more emotional support is needed when experiencing stress and confusion, and that expressing one’s emotions is more helpful [5].

### Interested topics by pregnancy stage

In this study, the participants were asked to describe the topics that they were interested in over the past two weeks. The answers were classified and compared according to pregnancy stage.

Pregnancy-related symptoms were ranked first regardless of the stage of pregnancy. Unlike other health problems, pregnancy symptoms include both normal- and high-risk symptoms. It is very difficult for first-time pregnant women to distinguish whether their symptoms are normal, leading to very high anxiety levels. A large amount of experience-based information on social media is needed to feel relieved.

Compared to the first and second trimesters of pregnancy with a variety of curious topics, the third trimester showed a tendency to focus on a few topics directly related to childbirth. Mood related topics appeared in the first trimester and post-partum stage. It is thus possible to identify the period during which emotional support of pregnant women is most needed.

A high interest in postpartum care, diet, and medication is interpreted as a characteristic of Korean or Asian women. It is well known that Asian women’s beliefs in postpartum care differ from those of Western women [22]. Especially in Korea, postpartum care methods such as postpartum care centers and postpartum caregivers are very popular [23]. Therefore, there was high interest in sharing experiences about what kind of postpartum care worked well across all stages of pregnancy. Compared to the topics of interest in Baker and Yang [12] study, which showed “pregnancy” as 76.9%, “childbirth” as 39.3%, and “healthy living” as 26.5%, and in the study by Sharifi, Amiri-Farahani [24], which showed the highest score with “fetal care” and “physical health”, in this study, “interest in health living” was high (48.7%) and there were many topics about what to eat and possible medication use, especially in early pregnancy. Lee, Park [13] reported that Koreans attach great importance to what they should eat during disease management. A study conducted in Germany [15] found that women had higher health and nutritional awareness than men. These findings can explain the interest in diet among pregnant Korean women. A strength of this study is that it compares the topics of interest by stage and thus the results of this study can be used to deliver high-quality information appropriate for each pregnancy stage.

### Satisfaction with informational support or emotional support by topic

Informational support was high in “daily life” and “antenatal screening tests,” while emotional support was high in topics related to family stories and emotions during pregnancy.

One interesting result was that the emotional support effect was high for the family topic in this study. Similarly, in a study conducted in China [3], there were many mentions of husbands and mothers-in-law or mothers during pregnancy, which was interpreted as a different result from the US. Sharing these private or sensitive topics seems to give them fun or lessen their anger [4].

This result suggests the need to classify topics as those that either strengthen the reliability of information or strengthen the sharing of experiences and feelings.

### Practical implications

Practical implications based on the results are suggested as follows.

Pregnant women require a combination of information and emotional support, and the importance of reliability, promptness, and empathy varies depending on the topic. There are topics that require prompt answers, topics that require the accumulation of diverse and detailed personal experiences, and topics that require expertise-based comments. Therefore, it is necessary to use different content or platforms. For topics that require more emotional support, a platform with many participant comments or replies would be useful.

Second, it is necessary to consider how to utilize the advantages of social media to provide reliable health information from health professionals. Influential social media can disseminate as much expertise-based information as possible to many pregnant women. However, it should be monitored to ensure that the information does not become distorted.

Third, experience-based information accumulated thus far through social media can be used to grasp the needs or interests of pregnant women. Just as a search engine detects local epidemics using search engine query data, posts on social media can be used as important data [25].

### Limitation

In this study, it is difficult to generalize the results because it involved a limited number of participants in Korea through snowball sampling. The high rates of online and social media use among Korean women should be considered. It is also necessary to understand the cultural background of Asian women's pregnancies and childbirths. Therefore, it is desirable to conduct a study targeting a wider area and larger number of participants in the future. This enables the effective delivery of reliable health content using social media’s growing popularity.

## Conclusions

This study confirmed that many pregnant women obtain health information through social media and feel satisfied with the information and emotional support provided thereby. The health topics they wanted were slightly different according to their stage of pregnancy, and the need for information or emotional support differed according to health topics. Based on this result, it is possible to provide higher-quality support using varying content or platforms for pregnant women according to their stage of pregnancy or topics of interest. Social media that can disseminate expert information and accumulate the experiences of women in similar situations can serve as a good source of health information for pregnant women.

## Supplementary Information

**Additional file 1: Supplementray Table 1.** Dataset of social media utilization of pregnancy women

## Data Availability

All data generated or analysed during this study are included in this published article and its supplementary information files.

## Abbreviations

eHEALS: EHealth Literacy Scale
KeHEALS: Korean eHealth Literacy Scale
